# Extraction of Geriatric Syndromes From Electronic Health Record Clinical Notes: Assessment of Statistical Natural Language Processing Methods

**DOI:** 10.2196/13039

**Published:** 2019-03-26

**Authors:** Tao Chen, Mark Dredze, Jonathan P Weiner, Leilani Hernandez, Joe Kimura, Hadi Kharrazi

**Affiliations:** 1 Center for Language and Speech Processing Whiting School of Engineering Johns Hopkins University Baltimore, MD United States; 2 Department of Computer Science Whiting School of Engineering Johns Hopkins University Baltimore, MD United States; 3 Center for Population Health IT Bloomberg School of Public Health Johns Hopkins University Baltimore, MD United States; 4 Academic Institute Atrius Health Boston, MA United States; 5 Division of Health Sciences Informatics School of Medicine Johns Hopkins University Baltimore, MD United States

**Keywords:** geriatrics, clinical notes, natural language processing, information extraction, conditional random fields

## Abstract

**Background:**

Geriatric syndromes in older adults are associated with adverse outcomes. However, despite being reported in clinical notes, these syndromes are often poorly captured by diagnostic codes in the structured fields of electronic health records (EHRs) or administrative records.

**Objective:**

We aim to automatically determine if a patient has any geriatric syndromes by mining the free text of associated EHR clinical notes. We assessed which statistical natural language processing (NLP) techniques are most effective.

**Methods:**

We applied conditional random fields (CRFs), a widely used machine learning algorithm, to identify each of 10 geriatric syndrome constructs in a clinical note. We assessed three sets of features and attributes for CRF operations: a base set, enhanced token, and contextual features. We trained the CRF on 3901 manually annotated notes from 85 patients, tuned the CRF on a validation set of 50 patients, and evaluated it on 50 held-out test patients. These notes were from a group of US Medicare patients over 65 years of age enrolled in a Medicare Advantage Health Maintenance Organization and cared for by a large group practice in Massachusetts.

**Results:**

A final feature set was formed through comprehensive feature ablation experiments. The final CRF model performed well at patient-level determination (macroaverage F1=0.834, microaverage F1=0.851); however, performance varied by construct. For example, at phrase-partial evaluation, the CRF model worked well on constructs such as absence of fecal control (F1=0.857) and vision impairment (F1=0.798) but poorly on malnutrition (F1=0.155), weight loss (F1=0.394), and severe urinary control issues (F1=0.532). Errors were primarily due to previously unobserved words (ie, out-of-vocabulary) and a lack of context.

**Conclusions:**

This study shows that statistical NLP can be used to identify geriatric syndromes from EHR-extracted clinical notes. This creates new opportunities to identify patients with geriatric syndromes and study their health outcomes.

## Introduction

Geriatric syndromes encompass multifactorial health conditions in older adults that generally do not fit into specific disease categories [1,2]. Geriatric syndromes, such as walking difficulty, falls, and incontinence, are often associated with adverse outcomes, such as morbidity, mortality, hospitalizations, and nursing home admissions [3]. Measuring geriatric syndromes at a population level is of great interest to health care providers and researchers to determine correlates of adverse outcomes. Hazra et al [4] contrasted health status, as measured by geriatric syndromes, of men and women aged 100 years or older. In addition, Anzaldi et al [5] measured prevalence of geriatric syndromes among individuals aged 65 years or older who had a mention of frailty in their clinical notes.

However, the multifactorial nature of geriatric syndromes complicates commonly accepted definitions for the recognition, diagnosis, and coding of these syndromes [1]. As a consequence, geriatric syndromes are often poorly captured by diagnostic codes (eg, International Classification of Diseases [ICD]9/10 codes) in the structured field of electronic health records (EHRs) [5], thus limiting research opportunities. Yet the unstructured clinical note (ie, free text) of EHRs contains rich information that describes geriatric syndromes. Considering the high adoption rate of EHRs among health care providers, unlocking information from clinical notes would yield a valuable resource for geriatric research and patient- and population-level interventions.

We propose a method to automatically identify patients exhibiting geriatric syndromes by analyzing text in clinical notes. We focus on 10 geriatric syndrome constructs: falls, malnutrition, dementia, severe urinary control issues, absence of fecal control, visual impairment, walking difficulty, pressure ulcers, lack of social support, and weight loss. We present methods based on natural language processing (NLP), specifically information extraction, that identify spans of text (ie, phrases) that attest to a geriatric syndrome. Previously, such clinical NLP has been leveraged to extract medical entities and concepts [6], such as disorder entity [7,8], medication [9], adverse drug reaction [10], smoking status [11], and risk [12].

Clinical NLP techniques can be roughly divided into two types: rule-based systems and machine learning (ie, statistical) techniques. Rule-based systems, which have long been the norm in the clinical setting, rely on manual definition of rules (eg, regular expressions) that identify phrases of interest in notes. For example, Anzaldi et al [5] and Kharrazi et al [13] developed a set of regular expressions to extract 10 geriatric syndrome constructs from clinical notes. The performance of rule-based approaches, however, requires significant domain expertise and is limited by the inflexibility of rule-based pattern matching. Therefore, statistical NLP methods based on machine learning techniques have long become the norm in the NLP community, with both academic research and industry systems relying almost exclusively on these methods [6]. Statistical methods rely on the construction of a manually annotated dataset to train a machine learning model; the trained model is then applied to extract entities and concepts from unannotated notes.

We propose to extract geriatric syndrome constructs from clinical notes using the conditional random field (CRF), a supervised statistical machine learning model that shows superior performance in many general and clinical information extraction tasks [3,7,8]. However, unlike most work in clinical NLP that focuses on well-defined entities and concepts (eg, based on Unified Medical Language System concepts), geriatric syndromes are often open-ended without clear definitions. For instance, the text spans of the geriatric syndrome constructs are much longer (ie, average length of 3.3 words) than well-defined entities and concepts (eg, average length of disorder entities are 1.8 tokens [ie, words]) [14]. Additionally, a lack of a clear coding standard for some of the geriatric syndrome constructs [1] means annotations are inconsistent, often including or excluding adjacent terms in the annotated construct mention. These challenges call into question the ability to develop a statistical NLP system for identifying patients with geriatric syndromes.

The objective of this paper is to explore the efficacy of a CRF model and various feature (ie, attribute) sets for the identification and classification of geriatric syndromes from clinical text. We consider the use of three feature sets: a base feature set, a token-enhanced set, and a set that includes contextual information. We evaluate the effectiveness of the model at identifying specific mentions (ie, phrases) as well as the overall ability to associate a construct with a patient (ie, aggregation over multiple notes). We report results on each of the 10 individual constructs and examine the factors that cause the accuracy of the trained model to vary over these constructs.

## Methods

We begin with a description of the dataset followed by the clinical NLP model used to identify geriatric syndrome constructs. We then describe our data and experimental setting.

### Dataset

We used anonymized EHR data shared with us by a large multispecialty medical group from New England, United States, for a cohort of elderly patients enrolled in a regional Medicare Advantage Health Maintenance Organization. We utilized a cohort of 18,341 members aged 65 years or older who received health insurance coverage between 2011 and 2013 and were assigned to this medical group as their primary source of medical care from this health plan. Clinical notes are documentations associated with patients’ encounters; the top five encounter types were phone calls (37.8%), office visits (30.2%), refill (11.6%), letter (3.2%), and notation (2.6%). All data were stored on a secured network approved by the Institutional Review Board (IRB) of Johns Hopkins University School of Public Health (IRB number 6196).

For the analysis reported here, we randomly selected a small sample of 185 patients from the above cohort and included all of their unstructured clinical notes, resulting in a dataset of 8442 notes. These notes were manually annotated by three physicians to indicate mentions of the 10 constructs. All the physicians went through a training phase and coded a similar text to ensure an acceptable agreement could be reached before conducting the annotation. Each note was annotated by one of the physicians. Table 1 shows a few example annotations. The clinical notes were structured into sections indicated by a section header (eg, *Patient Medical History* and *Current Outpatient Prescriptions*). We segmented the notes by section using a list of keywords [15] and applied sentence segmentation and tokenization using the clinical Text Analysis Knowledge Extraction System (cTAKES) (Apache) [16], a clinical text-processing tool.

### Clinical Natural Language Processing Algorithm

We modeled the construct identification and classification as a sequence labeling task. In a sequence labeling task, the model identifies and types spans of text according to established guidelines. Common sequence labeling tasks include part-of-speech taggings (ie, identifying each word as a noun, verb, or adjective) and named entity recognition (ie, identifying spans of text that refer to people, organizations, and locations by name). CRFs [17] are widely used statistical models for sequence labeling tasks in both traditional NLP [18] and clinical NLP [6-8]. In addition, CRFs were used by several validated systems in clinical note information extraction shared tasks [7,8]. We utilized the linear-chain CRF implementation from the CRFSuite software package developed by Naoaki Okazaki [19] for our work.

As a supervised machine learning algorithm, the CRF estimates (ie, learns) model parameters based on an annotated dataset (ie, training set). The trained model can then predict the labels of sequences without annotation. A key input to the model is a set of features: attributes of the input upon which the CRF builds a model and estimates parameters. Feature choices are a critical factor in determining the resulting performance of the model [17].

We designed and evaluated three sets of features to extract from the clinical note. These features capture basic information of the tokens (ie, words), enhanced information of the token, and the global context:

1. Basic Features: This set of features includes the lowercase of the token, the part-of-speech tag of the token, as identified by cTAKES, and three orthographic features that indicate whether the token is numeric, in uppercase, or in title case. In addition, we captured the local context of the token by incorporating these features from the previous and next token. In total, each token has 18 feature types.
2. Enhanced Token Features: This set of features captures additional features about the token. Many studies show that the stem of the token is a useful feature for information extraction tasks [20], thus we encoded it as another feature and explored how these features perform in the EHR domain. We used another two orthographic features to indicate whether the token is an ICD9 code [21] (hereafter, Is-ICD9-Code; we could not evaluate ICD10 code, as our dataset predates the release of ICD10 code in 2015) and whether the token is a medical measurement unit (eg, kg or mL; hereafter, Is-Medical-Unit). To obtain these two features, we compared the token against the ICD9 codes [21] and common medical measurement units and encoded them as two binary features. The mention of a construct may not necessarily indicate the associated geriatric syndrome for the patient. The mention may be negated or reflect uncertainty in the diagnosis or could refer to another individual (eg, patient's family member). To identify such cases, we used cTAKES to obtain three attributes: negation, uncertainty, and subject of the construct entity mention. We encoded these as three features for each token (hereafter, Entity-Attributes).
3. Global Context: Our final feature set captures high-level context based on characteristics of the patient or clinical note. We first consider the section in which a token appears, as some section headers may suggest the mention does not refer to the patient (eg, *Family History*). Our second global context feature uses ICD9 codes mentioned in the text. Prior work [5,13] identified a list of 295 ICD9 codes that are indicative of the 10 constructs; Table 2 details the number of ICD9 codes per construct. We leveraged this list to encode 10 binary features to indicate the mapping of a token to the 10 constructs (hereafter, ICD9-Annotation). That is, one binary feature that corresponds to the construct will be true when the token is in the list; otherwise, all 10 of the binary features are false. Alternatively, we utilized this list to modify the prediction from the CRF as a postprocessing step. When the token is present in the list, we altered its label to reflect the construct label from the list, even if the CRF failed to identify a corresponding construct mention (hereafter, ICD9-Annotation-Post).

### Experimental Setting

Following standard evaluation conventions, we randomly split the 185 annotated patients into a training set (N=85), validation set (N=50), and test set (N=50). From Table 3, we see the 10 constructs have skewed distributions. In the training set, the two most dominant constructs were walking difficulty and lack of social support, which were present in 66% (56/85) and 62% (53/85) of patients, respectively; the two rare constructs, malnutrition and pressure ulcers, were present in only 9% (8/85) and 11% (9/85) of patients, respectively. We estimated model parameters on the training set, tuned the hyperparameters of the training algorithm, chose features to include on the validation set, and evaluated our final trained model on the held-out test set. This evaluation procedure ensures that test set performance reflects real-world system accuracy, as choices of algorithm design and parameter estimates are made blind to the test set data.

**Table 1:** Example sentences from clinical notes that contain a construct: annotated construct phrases are italicized.

Construct	Example sentence from clinical notes (verbatim)
Absence of fecal control	She has also been experiencing urinary incontinence and a few *episodes of fecal incontinence* too.
Dementia	Patient *has dementia* and daughter feels as though it has worsened since Labor day.
Falls	She suffered *a fall* this past Tuesday and then was complaining of left shoulder pain.
Weight loss	Sed rate had been mildly elevated except the last one over 70 but in setting of acute illness and *weight loss*.
Malnutrition	*Inadequate energy intake* as evidenced by weight loss.
Pressure ulcers	She has 2 *intragluteal decubitus*.
Lack of social support	She *is alone* at home much of the day.
Severe urinary control issues	She *has a suprapubic catheter* in (placed under interventional radiology at) because she was having pain on urination.
Visual impairment	Has been seen by vision rehab and *is registered with of blind*.
Walking difficulty	*Ambulates slowly*, uses vital signs as above.

**Table 2:** Statistics related to the 10 constructs.

Construct	Number of ICD9^a^ codes that indicate a construct (n)	Average number of tokens per construct	Average number of mentions per patient in the test set	Perplexity on test set^b^
Absence of fecal control	2	2.98	2.67	11.30
Dementia	58	2.76	13.00	26.28
Falls	45	3.37	9.04	57.68
Weight loss	15	3.01	13.53	33.80
Malnutrition	26	2.04	13.92	100.64
Pressure ulcers	35	3.48	5.67	66.90
Lack of social support	14	4.03	15.23	29.96
Severe urinary control issues	14	2.94	13.71	117.48
Visual impairment	55	3.62	9.31	57.68
Walking difficulty	31	3.43	12.59	84.27

^a^ICD9: International Classification of Diseases 9.

^b^Perplexity is computed on the test set based on the construct-specific language model trained on the training set: detailed in the Error Analysis section.

We performed feature ablation experiments on the validation set to assess the effectiveness of the proposed features. In each experiment, we trained the CRF on the training set using basic features and one or more features from the proposed feature sets, evaluating the trained model on the validation set. For each experiment, we used the validation set to tune model hyperparameters in a grid search manner. Example hyperparameters are L2 regularizer and maximum number of iterations; the learning rate of stochastic gradient descent (SGD) is automatically determined by CRFSuite. We also assessed both the limited-memory Broyden-Fletcher-Goldfarb-Shanno (L-BFGS) algorithm and SGD optimization methods, selecting SGD as our final training algorithm for its better performance. Finally, we trained the model with the best feature combination and hyperparameter settings and reported the final performance on the test set.

We consider four different evaluation metrics for our CRF. The most restrictive evaluation measure is phrase-exact, in which we mark a prediction as correct only if the extracted and identified phrase exactly matched the labeled phrase. Under this metric, for the sentence “This patient walks with a walker,” an answer would only be correct if it identified the phrase “with a walker” (ie, the provided annotation) as walking difficulty but not if it selected “walks with a walker.” While this type of evaluation is standard in many information extraction tasks, it is too strict considering that our goal is to associate a geriatric syndrome with the patient. On the other hand, we also observed our three annotators sometimes exhibiting inconsistency in including or excluding unimportant words (eg, prepositions and verbs) in the annotated phrase (eg, “with a walker,” “walks with a walker,” or “walker”). Therefore, we consider a partial matching metric (ie, phrase-partial), which marks a prediction as correct if the predicted phrase overlaps with the manual annotation. In this setting, the above prediction for walking difficulty would be correct. Such partial matching has also been adopted in other clinical information extraction tasks [7,8].

**Table 3:** The construct and nonconstruct distribution among three datasets based on manual annotation.

Construct	Training set (3901 notes)		Validation set (1739 notes)		Test set (2802 notes)	
	Token^a^ (N=1,083,670), n (%)	Patient^b^ (N=85), n (%)	Token (N=435,851), n (%)	Patient (N=50), n (%)	Token (N=638,694), n (%)	Patient (N=50), n (%)
Absence of fecal control	126 (0.01)	12 (14)	126 (0.03)	4 (8)	34 (0.01)	3 (6)
Dementia	631 (0.06)	15 (18)	276 (0.06)	9 (18)	403 (0.06)	10 (20)
Falls	1419 (0.13)	37 (44)	293 (0.07)	21 (42)	748 (0.12)	23 (46)
Weight loss	365 (0.03)	21 (25)	263 (0.06)	14 (28)	752 (0.12)	19 (38)
Malnutrition	115 (0.01)	8 (9)	82 (0.02)	5 (10)	312 (0.05)	12 (24)
Pressure ulcers	308 (0.03)	9 (11)	18 (0.00)	4 (8)	126 (0.02)	6 (12)
Lack of social support	2026 (0.19)	53 (62)	1410 (0.32)	30 (60)	1691 (0.26)	30 (60)
Severe urinary control issues	694 (0.06)	16 (19)	81 (0.02)	4 (8)	323 (0.05)	7 (14)
Visual impairment	324 (0.03)	16 (19)	141 (0.03)	6 (12)	395 (0.06)	13 (26)
Walking difficulty	2253 (0.21)	56 (66)	1315 (0.30)	26 (52)	1423 (0.22)	34 (68)
Nonconstruct	1,075,409 (99.24)	85 (100)	431,846 (99.08)	50 (100)	632,487 (99.03)	50 (100)

^a^Denotes the number of tokens in the dataset that were labeled as certain constructs.

^b^Denotes the number of patients in the dataset who were identified containing certain constructs.

Since our goal was to associate constructs with patients, we considered two additional metrics. First, we identified the prediction of a construct as correct if that construct appeared anywhere in the clinical note (ie, note-level), which may occur when an annotator missed a construct mention. We also considered a patient-level evaluation, in which we marked a prediction as correct if the patient had the associated construct (eg, an annotator identified it somewhere in an associated note).

We computed the popular information extraction metrics of precision (ie, true positive/[true positive + false positive]), recall (ie, true positive/[true positive + false negative]), and their harmonic mean (F1 or F measure). These metrics are related to sensitivity (ie, true positive rate) and specificity (ie, true negative rate). We provide these metrics for each of the four evaluation types and report model performance on each of the 10 constructs. We also report micro- and macroaveraged results, where microaveraging computes the average over every construct mention and macroaveraging gives equal weight to every construct. The difference reflects the variations of prevalence among the constructs. For all validation set choices, we used microaveraged F1 on the phrase-partial matching metric.

### Error Analysis

To gain more insights, we performed an in-depth analysis on the system’s errors. We quantified the chances that the CRF model would confuse the mention of one construct with another. Additionally, we trained 10 construct-specific bigram language models (ie, a probability distribution to estimate the relative likelihood of text) using the construct’s mention texts from the training set. We then computed the perplexity (ie, a measurement of how well a probability distribution predicts a sample) of each construct language model on mentions of the construct in the test set. In short, the perplexity captures how “surprised” the model would be by a construct reference in the test data based on how the construct was referenced in the training data.

### Ethical Considerations

This study was approved by the IRB of Johns Hopkins University School of Public Health (IRB number 6196). Participant consent was not required as data was deidentified prior to analysis.

## Results

We measured the micro- and macroaverage phrase-partial results of models with optimal hyperparameters on the validation data (see Table 4). Using the basic feature set alone, the CRF achieved a macroaverage F1 score of 0.583 and a microaverage F1 score of 0.727. Most additional features improved the F1 score. Of these, the most effective single feature was the stem, which improved the macroaverage F1 score by 0.103, a relative improvement of 17.7%, and improved the microaverage F1 score by 0.033, a relative improvement of 4.5%, when compared to the basic model using basic features. The two exceptions were token-enhanced features: Is-ICD9-Code and Is-Medical-Unit. Though the two features did not improve the F1 score, they did increase the microaverage precision from 0.93 to 0.959 (Is-ICD9-Code) and 0.948 (Is-Medical-Unit). This suggests that they could still contribute to overall improvements when combined with other features focused on improving recall.

**Table 4:** Phrase-partial evaluation on the validation set.

Feature set and features		*P* value^a^	Macroaverage				Microaverage		
			Precision	Recall	F1	Precision		Recall	F1
**Basic features**									
	Basic	N/A^b^	0.828	0.450	0.583	0.930		0.597	0.727
**Enhanced token features**									
	B^c^+Is-ICD9^d^-Code	<.001	0.874	0.472	0.613	0.887		0.640	0.744
	B+Is-Medical-Unit	<.001	0.828	0.402	0.541	0.959		0.538	0.689
	B+Entity-Attributes	<.001	0.823	0.398	0.537	0.948		0.528	0.678
	B+Stem	.03	0.856	0.572	0.686	0.864		0.678	0.760
**Contextual features**									
	B+Section	<.001	0.783	0.544	0.642	0.874		0.682	0.766
	B+ICD9-Annotation	<.001	0.888	0.462	0.608	0.928		0.598	0.727
	B+ICD9-Annotation-Post	<.001	0.823	0.478	0.605	0.912		0.604	0.727
**Combination (B+Enhanced+Context)**									
	B+all Enhanced (C^e^+U^f^+E^g^+S^h^)+all Context (T^i^+A^j^+AP^k^)	<.001	0.793	0.633	0.704	0.757		0.714	0.735
	B+Enhanced (C+E+S)+all Context (T+A+AP)	<.001	0.837	0.483	0.613	0.895		0.546	0.678
	B+Enhanced (C+E+S)+Context (A+AP)	<.001	0.874	0.529	0.659	0.906		0.630	0.743
	*B+all Enhanced (C+U+E+S)+Context (A+AP)* ^l^	*<.001*	*0.862*	*0.567*	*0.684*	*0.880*		*0.681*	*0.768*
	B+Enhanced (C+S)+Context (A+AP)	<.001	0.799	0.509	0.622	0.896		0.616	0.730
**Non-CRF^m^** **model**									
	Only uses annotated ICD9 codes as a rule to identify constructs	<.001	0.803	0.139	0.236	0.885		0.059	0.111

^a^We conducted McNemar's test to measure the difference between the results of using basic features and other features.

^b^N/A: not applicable.

^c^B: basic.

^d^ICD9: International Classification of Diseases 9.

^e^C: Is-ICD9-Code.

^f^U: Is-Medical-Unit.

^g^E: Entity-Attributes.

^h^S: stem.

^i^T: section.

^j^A: ICD9-Annotation.

^k^AP: ICD9-Annotation-Post.

^l^The best-performing model is italicized.

^m^CRF: conditional random field.

In contrast, the section feature leads to a significant improvement on recall but lowers the precision by a large margin. Therefore, we further evaluated combinations of these three features and other features on the validation set. The best-performing model (L2 regularizer=0.2, maximum number of iterations=100) on validation data was trained with all the features except section and achieved a macroaverage F1 score of 0.684 and a microaverage F1 score of 0.768. This model significantly (*P*<.001 via McNemar's test) outperformed the basic model with a macroaverage F1 score of 0.101, a relative improvement of 17.3%, and a microaverage F1 score of 0.041, a relative improvement of 8.8%. Additionally, we implemented a simple rule-based method that only uses the annotated ICD9 codes to identify constructs in a sentence. As expected, this method achieved a very low recall (0.139 at macroaverage and 0.059 at microaverage) and F1 score (0.236 at macroaverage and 0.111 at microaverage), which further validates that geriatric syndromes are poorly captured by diagnosis code. This also demonstrates the importance of developing models to identify constructs by mining unstructured text.

**Figure 1:**
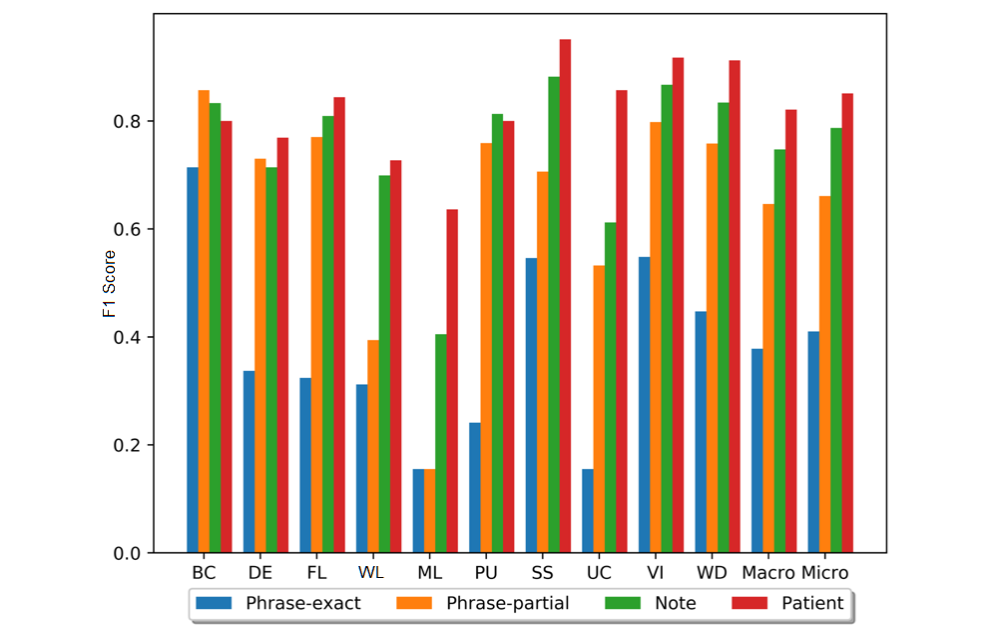
The F1 scores of the best-performing model on the test set. BC: absence of fecal control; DE: dementia; FL: fall;, WL: weight loss; ML: malnutrition; PU: pressure ulcers; SS: lack of social support; UC: severe urinary control issues; VI: visual impairment; WD: walking difficulty; Macro: macroaverage; Micro: microaverage.

We evaluated this best-performing CRF model on the test set and report per-construct results in addition to overall averages (see Table 5 and Figure 1). The CRF obtained macroaverage F1 scores of 0.394 for phrase-exact, 0.666 for phrase-partial, 0.759 for note, and 0.834 for patient. Microaverage F1 scores were 0.410 for phrase-exact, 0.661 for phrase-partial, 0.787 for note, and 0.851 for patient. Across all constructs, precision was higher than recall, meaning that the model favored accurate predictions over coverage of construct mentions. By relying on this high-precision approach and the repetition of construct mentions in patient’s clinical notes (see Table 2) (eg, the lack of social support was mentioned, on average, 15.23 times per patient), our model obtained a much higher performance on patient than on the other three evaluations—phrase-exact, phrase-partial, and note.

Performance varied widely for different constructs (see Table 5 and Figure 1). On a phrase-partial analysis, seven constructs generated an F1 score of over 0.7, of which absence of fecal control was the best (0.857), while the three worst constructs—malnutrition, weight loss, and severe urinary control issues—obtained scores of 0.155, 0.394, and 0.532, respectively. To understand the F1 variance across the constructs, we performed an in-depth error analysis of system errors. Overall, 97.0% of errors were caused by missing the constructs; the model seldomly confused mentions of one construct for another. One primary cause of low recall was the limited training instances, which limited the variations observed during training. The training set contains 3901 notes from 85 patients, but only 0.76% of tokens indicated any of the 10 geriatric syndromes. For the three poorly performing constructs, we found that both malnutrition and severe urinary control issues had a high out-of-vocabulary (OOV) rate in the test set (ie, many of the words used to refer to these constructs were unobserved during training).

We measured the perplexity scores for each construct (see Table 2). This score measures how unprepared the model would be by the construct’s mention in the test set. Malnutrition (100.64) and severe urinary control issues (117.48) showed a much higher perplexity score than the other constructs, confirming higher OOV rates as compared to the other constructs. However, the poor performance of weight loss was primarily caused by confusion between intentional weight loss (ie, nonconstruct; patients who are overweight and thus trying to lose weight) and unintentional weight loss (ie, our construct). Since intentional weight loss is dominant, the CRF identified incidents of weight loss as nonconstruct, yielding a very low recall (0.272) and F1 score (0.394).

**Table 5:** The evaluation results of the best-performing model on the test set.

Construct	Phrase-exact				Phrase-partial			Note			Patient		
	Precision	Recall	F1	Precision		Recall	F1	Precision	Recall	F1	Precision	Recall	F1
Absence of fecal control	0.833	0.625	0.714	1		0.750	0.857	1	0.714	0.833	1	0.667	0.800
Dementia	0.324	0.350	0.337	0.703		0.759	0.730	0.604	0.873	0.714	0.625	1	0.769
Falls	0.387	0.279	0.324	0.942		0.651	0.770	0.926	0.719	0.809	0.864	0.826	0.844
Weight loss	0.571	0.215	0.312	0.714		0.272	0.394	0.866	0.586	0.699	0.857	0.632	0.727
Malnutrition	0.577	0.090	0.155	0.577		0.090	0.155	0.680	0.288	0.405	0.700	0.583	0.636
Pressure ulcers	0.304	0.200	0.241	0.957		0.629	0.759	0.929	0.722	0.813	1	0.667	0.800
Lack of social support	0.551	0.541	0.546	0.707		0.706	0.706	0.923	0.845	0.882	0.935	0.967	0.951
Severe urinary control issues	0.207	0.124	0.155	0.690		0.433	0.532	0.682	0.556	0.612	0.857	0.857	0.857
Visual impairment	0.687	0.456	0.548	1		0.664	0.798	1	0.765	0.867	1	0.846	0.917
Walking difficulty	0.517	0.394	0.447	0.842		0.689	0.758	0.894	0.781	0.834	0.912	0.912	0.912
Macroaverage	0.496	0.327	0.394	0.813		0.564	0.666	0.850	0.685	0.759	0.875	0.796	0.834
Microaverage	0.493	0.351	0.410	0.785		0.571	0.661	0.806	0.726	0.787	0.868	0.834	0.851

## Discussion

### Principal Findings

Geriatric syndromes are often not captured in EHRs’ structured fields, which requires examining of EHRs’ free text to identify older adults with such syndromes [5,13]. We applied NLP techniques to identify patients who have geriatric syndromes by analyzing their clinical notes. We trained CRF models with three sets of features to identify phrases that indicate geriatric syndromes and aggregated identified phrases to make patient-level determinations. Our best-performing model obtained a macroaverage F1 score of 0.834 and microaverage F1 score of 0.851 for identifying 10 geriatric syndrome constructs for patients. Our system identified most patients with geriatric syndromes by mining EHRs’ clinical notes and could support research on geriatric syndromes.

### Technical Challenges and Opportunities

Extracting and identifying geriatric syndromes is much more difficult than well-defined entity extraction. For instance, in the disorder entity extraction task of the 2013 Shared Annotated Resources/Conference and Labs of the Evaluation Forum (ShARe/CLEF) eHealth Evaluation Lab [22], a shared task focusing on clinical NLP, the best-performing system [23] also relied on CRF models but obtained much better results (phrase-partial F1=0.873) than did ours (macroaverage F1=0.666, microaverage F1=0.661). Our model heavily favors precision over recall (eg, macroaverage precision=0.813 vs macroaverage recall=0.564 at phrase-partial) and exhibits varied performance on different constructs; it works well on the absence of fecal control (F1=0.857), visual impairment (F1=0.798), and fall (F1=0.770), but poorly on malnutrition (F1=0.155), weight loss (F1=0.394), and severe urinary control issues (F1=0.532). These variations are primarily caused by the high OOV rates of malnutrition and severe urinary control issues as well as the confusion between intentional weight loss (ie, nonconstruct) and unintentional weight loss (ie, our construct). These challenges may limit statistical NLP models in geriatric syndrome research since the models will miss a large portion of patients with malnutrition, severe urinary control issues, and weight loss.

Our error analysis suggests several directions of future work. We can lower OOV rates by obtaining additional annotated clinical notes. However, manual annotation is time-consuming and costly (eg, it took approximately 240 person-hours in total for the three experts to annotate 185 patients); we thus prefer other solutions. One approach would be to generalize representations away from lexical forms, replacing words or phrases with embeddings. Embeddings are a form of high-dimensional dense vector representation where similar words or phrases tend to be close to each other. Such embeddings could be trained with a large number of unlabeled notes in an unsupervised fashion [24,25], which have shown to be effective in clinical NLP tasks [26,27]. Since we can learn embeddings even for words unseen in training, this may mitigate the OOV problem. In addition, we should identify the larger context of the mention to differentiate intentional and unintentional weight loss. For example, if the note discusses obesity, it is a strong indicator that the weight loss is intentional. This contextual information may be reflected in the diagnostic codes in the structured field of EHRs or the larger context in the free text. We could incorporate the contextual information into the extraction model through the use of learned representations of the context (ie, embeddings). Finally, recent interest in deep learning model architectures [28] have shown promise on clinical data [29,30]. These models may provide added benefits over CRFs.

### Clinical and Population Health Implications

Our work has implications for managing older adult patients by enabling clinicians and researchers to identify a broader set of patients with geriatric syndromes using EHRs’ free text. For example, a wider identification of geriatric syndromes enables clinicians to adjust interventions and provides researchers the opportunity to expand study cohorts [13]. However, extracting geriatric syndromes from clinical notes may require dealing with multiple EHR issues, such as lack of data-quality specifications for EHRs and increased rate of missing data over time [31], challenges with incorporating questionnaires within the EHRs’ architecture (eg, geriatric frailty questionnaires) [32], and variation of EHR use and maturation across different health delivery systems [33].

Population health management efforts are increasingly using EHR data for risk stratification of patient populations [34-38]. Our model increases the coverage of risk stratification models developed specifically for an older adult population. Identification of new cases of geriatric syndromes will enable care coordinators and case managers to better manage patient populations [34], which can lead to more streamlined and efficient workflows. Furthermore, our work has implications for extracting nonclinical information, such as social determinants of health (SDH)—an open-ended construct similar to geriatric syndromes—from EHRs’ free text. SDH is a combination of lifestyle, behavioral, social, economic, and environmental factors that are powerful drivers of health and well-being [39]. Similar to geriatric syndromes, SDH are poorly captured by diagnostic codes in EHRs [40-42]. More broadly, our success with NLP techniques for geriatric syndromes suggests that other areas that actively use SDH information to bridge the gap between population and public health may similarly benefit from EHRs’ free text and NLP techniques [43-46].

### Limitations

This work has several limitations. First, given the evolving concept of geriatric syndromes and their varied contextual information mentioned in clinical notes, and despite the rigorous training of the annotators, annotations were slightly inconsistent in including or excluding unrelated contextual wording (eg, including or excluding the location of a fall in addition to the event of a fall). Additionally, from error analysis, we also observed a few cases where our CRF model correctly identified the construct mentions but were missed by human annotators. These issues could be alleviated when soliciting multiple experts to repeatedly annotate the notes. Second, we only experimented with the 185 annotated patients, while our dataset contains a large portion of unlabeled notes (ie, 18,156 patients). It will be interesting to apply our CRF model to the unlabeled notes and see how these 10 geriatric syndromes distribute in the larger population. Third, we limited our study to 10 constructs of geriatric syndromes, but many other types of geriatric syndromes (eg, delirium and functional decline) exist. We plan to generalize our model to extract other geriatric syndromes when their annotations are available. Finally, we did not analyze the temporal patterns of the geriatric syndrome constructs. Future studies should investigate whether the temporal patterns of a construct, especially if deemed resolvable over time such as lack of social support, will be altered differently by different NLP solutions (eg, measuring temporal accuracy).

### Conclusions

We have presented an NLP solution for the automatic identification and classification of patients exhibiting geriatric syndromes by analyzing free text in patient clinical notes. We formulated the problem as an information extraction task and trained a CRF model to identify geriatric syndrome constructs from text. We presented enhanced features for this task and created a final system that obtains a microaverage F1 of 0.851 for patient-level determination of constructs. Our error analysis revealed that new words and a lack of context account for the worst-performing constructs. Future work should develop strategies that do not require additional training annotations to mitigate these errors.

## Abbreviations

A: ICD9-Annotation
AP: ICD9-Annotation-Post
B: basic
C: Is-ICD9-Code
CRF: conditional random field
cTAKES: clinical Text Analysis Knowledge Extraction System
E: Entity-Attributes
EHR: electronic health record
ICD: International Classification of Diseases
IRB: Institutional Review Board
L-BFGS: limited-memory Broyden-Fletcher-Goldfarb-Shanno
NLP: natural language processing
OOV: out-of-vocabulary
S: stem
SDH: social determinants of health
SGD: stochastic gradient descent
ShARe/CLEF: Shared Annotated Resources/Conference and Labs of the Evaluation Forum
T: section
U: Is-Medical-Unit
